# A NURSING HOME–BASED COVID-19-ONLY REHAB PROGRAM: VARIABLES RELATED TO SUCCESSFUL COMMUNITY DISCHARGE

**DOI:** 10.1093/geroni/igac059.1807

**Published:** 2022-12-20

**Authors:** Orah Burack, Joann Reinhardt, Wingyun Mak, Ruth Spinner

**Affiliations:** The New Jewish Home, New York, New York, United States; The New Jewish Home, New York, New York, United States; The New Jewish Home Research Institute on Aging, New York, New York, United States; The New Jewish Home, New York, New York, United States

## Abstract

Many COVID-19 patients continue to test positive for COVID-19 beyond the typical quarantine period. Persistent positive testing complicated hospital discharges to nursing home (NH) based rehabilitation facilities when (in May 2020) New York State (NYS) required a negative COVID result prior to NH admissions. To meet the needs of medically stable persistent positive patients, NYS approved 19 NHs as COVID-only facilities (COF). These facilities provided rehabilitation services and allowed hospitals to regain critical space for incoming acute care patients. In the present study we describe the (1) establishment of a 100 bed COF, (2) patients admitted to the COF and care provided, and (3) predictors of successful discharge to the community.Information on the establishment of the COF was obtained from interviews with NH leadership and clinical staff. Patient, treatment, and outcome data were obtained from the NH’s electronic health record. Of 319 COF admissions over four months, 54% were female and the mean age was 80 years (SD=10.56). 51% had cognitive impairment and the mean number of comorbidities was 6.75 (SD=2.47). All patients received physical and occupational therapy while 37% received speech therapy. Medical treatment included: anticoagulants (52%), oxygen (37%), antibiotics (35%), inhaler/nebulizers (31%), and oral steroids (15%). 59% of COF stays resulted in successful community discharges. This outcome was more likely for patients who had fewer comorbidities, less speech therapy, and did not require antibiotics. Implications for COVID care as well as leveraging NH potential during crisis will be discussed.

